# Rhizosphere soil properties of waxy sorghum under different row ratio configurations in waxy sorghum-soybean intercropping systems

**DOI:** 10.1371/journal.pone.0288076

**Published:** 2023-07-06

**Authors:** Mingbo Shao, Can Wang, Lingbo Zhou, Fangli Peng, Guobing Zhang, Jie Gao, Siyu Chen, Qiang Zhao

**Affiliations:** Institute of Upland Food Crops, Guizhou Academy of Agricultural Sciences, Guiyang, Guizhou, China; Central Research Institute for Dryland Agriculture, INDIA

## Abstract

To overcome the continuous planting obstacle and promote the sustainable production of waxy sorghum, a two-years field experiment was performed to determine the responses of waxy sorghum rhizosphere soil properties to different row ratio configurations in waxy sorghum-soybean intercropping systems. The treatments included five row ratio configurations, which were two rows of waxy sorghum intercropped with one row of soybean (2W1S), two rows of waxy sorghum intercropped with two rows of soybean (2W2S), three rows of waxy sorghum intercropped with one row of soybean (3W1S), three rows of waxy sorghum intercropped with two rows of soybean (3W2S), and three rows of waxy sorghum intercropped with three rows of soybean (3W3S), and sole cropping waxy sorghum (SW) was used as control. The nutrients, enzyme activities, and microbes of waxy sorghum rhizosphere soil were investigated at the jointing, anthesis, and maturity stages. Results showed that rhizosphere soil properties of waxy sorghum were significantly affected by row ratio configurations of waxy sorghum intercropped soybean. Among all treatments, the performances of rhizosphere soil nutrients contents, enzymes activities, and microbes contents were 2W1S > 3W1S > 3W2S > 3W3S > 2W2S > SW. Compared to SW treatment, the 2W1S treatment increased the organic matter, total N, total P, total K, gram-negative bacteria phospholipid fatty acids (PLFAs), and gram-positive bacteria PLFAs contents and catalase, polyphenol oxidase, and urease activities by 20.86%-25.67%, 34.33%-70.05%, 23.98%-33.83%, 44.12%-81.86%, 74.87%-194.32%, 81.59–136.59%, 91.44%-114.07%, 85.35%-146.91%, and 36.32%-63.94%, respectively. Likewise, the available N, available P, available K, total PLFAs, fungus PLFAs, actinomycetes PLFAs, and bacteria PLFAs contents under the 2W1S treatment were 1.53–2.41, 1.32–1.89, 1.82–2.05, 1.96–2.91, 3.59–4.44, 9.11–12.56, and 1.81–2.71 times than those of SW treatment, respectively. Further, the determining factors of soil microbes were total K, catalase, and polyphenol oxidase for total microbes, bacteria, and gram-negative bacteria, total P and available K for fungus, available N, available K, and polyphenol oxidase for actinomycetes, and total K and polyphenol oxidase for gram-positive bacteria. In conclusion, the 2W1S treatment was the optimal row ratio configuration of waxy sorghum intercropped with soybean, which can improve the rhizosphere soil quality and promote the sustainable production of waxy sorghum.

## Introduction

Sorghum [*Sorghum bicolor* (L.) Moench] is the fifth largest cereal crop second only to rice, wheat, corn, and barley, which is cultivated in many countries around the world [1]. It is an important miscellaneous grain crop in China and can be used for the human food, animal feed, and industrial raw materials [2, 3]. Waxy sorghum is the main raw material for brewing Moutai-flavor liquor and plays an important role in boosting rural revitalization of Guizhou province, China [4, 5]. According to the statistics, the annual demand of liquor factories for waxy sorghum planting area is about two hundred and fifty thousand hectares in Guizhou. However, due to the limited per capita arable land area, continuous planting of waxy sorghum has led to the deterioration of soil nutrients, unbalance of soil microbial community structure, intensification of diseases and pests, and reduction of yield and quality [6, 7]. Therefore, it is urgent to find a suitable planting pattern to overcome the continuous planting obstacle of waxy sorghum.

Intercropping refers to the planting method of two or more crop species simultaneously planted in the same field during the same growing season, which can improve the land productivity when compared with sole planting systems [8–10]. At present, explanations for the benefits of intercropping systems are mainly reflected in the following four aspects. Firstly, it can promote the soil improvement, especially in terms of the soil microbes. As an example, the tobacco intercropped with soybean significantly increased the amounts of bacteria and actinomycetes, while reducing the fungus amount in rhizosphere soil [11]. Secondly, it can use the complementary effect of different niche resources to promote the rooting behavior of crops. For instance, intercropping has been found to enhance root functional traits in 20 cm depth of soil [12]. Thirdly, intercropping can improve crops ventilation and light conditions through the allocation of tall and short crops, so as to increase the light utilization efficiency of crops. A typical example is oat intercropped with legumes enhanced chlorophyll content and net photosynthetic rate of oat and altered chlorophyll composition which contributed to the slower process of oat aging [13]. Fourthly, intercropping can use biodiversity to control pests and diseases. Ma et al. [14] presented an example for this aspect that faba bean intercropped with wheat significantly decreased the chocolate spot disease index and area under disease progress curve of faba bean.

Intercropping of cereals and legumes is a sustainable planting pattern with ecological and intensive characteristics, which can reduce the dependence of agricultural production on chemical nitrogen fertilizer and pesticides through nitrogen fixation of legumes [15–17]. Legumes provide an important strategy to alleviate the constraints caused by soil nitrogen limitations and improve crop productivity, which can fix atmospheric nitrogen to reduce soil erosion and improve the land use efficiency [18–20]. Such horticultural cultivation can also help in the reduction of drought and salt stresses resulting from bad land use efficiency [21]. Sorghum intercropped with soybean often obtains more compound yield and economic benefit than those of their sole crop because of its more efficient use of resources [22, 23]. Sorghum is a typical cereal and a C_4_ crop with high photosynthetic capacity, because it can use very low CO_2_ content in the intercellular space of leaves for photosynthesis [24]. Soybean is a legume and C_3_ crop which can enhance the efficient use of nitrogen by using its own nitrogen-fixing bacteria to fix nitrogen sources [25]. These bacteria can help in the improvement of plant growth and development, as well as the bioremediation of different types of pollutants [26]. Thus, sorghum-soybean intercropping may be an option for improving crop yield and quality.

Row ratio configuration is a very important management measure in the intercropping systems, which can change the field microclimate and regulate interspecies competition to affect yield formation of intercrops [27, 28]. Thus, appropriate row ratio configuration is essential to alleviate the competition among intercrops and improve the land use efficiency. Yin et al. [29] have shown that the ecological and economic benefits were significantly affected by waxy sorghum intercropped with soybean. Nevertheless, to our knowledge, the changes of waxy sorghum rhizosphere soil properties in the waxy sorghum-soybean intercropping systems have not been reported, especially regarding row ratio configuration. Therefore, a two-years field experiment was performed to determine the responses of waxy sorghum rhizosphere soil properties to different row ratio configurations in waxy sorghum-soybean intercropping systems.

## Materials and methods

### Experimental sites and experimental design

In 2019 and 2020, the field experiments were performed at the Guiyang test base of Guizhou Academy of Agricultural Sciences. Waxy sorghum cultivar “Qiangao-7” and soybean cultivar “Qiandou-7” were used in this study. The treatments included five row ratio configurations of waxy sorghum intercropped soybean, which were two rows of waxy sorghum intercropped with one row of soybean (2W1S), two rows of waxy sorghum intercropped with two rows of soybean (2W2S), three rows of waxy sorghum intercropped with one row of soybean (3W1S), three rows of waxy sorghum intercropped with two rows of soybean (3W2S), and three rows of waxy sorghum intercropped with three rows of soybean (3W3S), and sole cropping waxy sorghum (SW) was used as control. All treatments were arranged in a randomized complete block arrangement with three replicates. Detailed descriptions of experimental site, experimental design, and various field management practices were presented in our previous studies [30, 31].

### Rhizosphere soil sampling

At the jointing, anthesis, and maturity stages, three planting holes of waxy sorghum were randomly selected in the middle strip of each plot, and roots were dug up using a small hoe. The loose surface soil on the roots was shaken off gently and rhizosphere soil which tightly adhered to roots was collected carefully with a brush. Rhizosphere soils from waxy sorghum plants in each plot were mixed to make a composite sample and sieved through a 2 mm mesh. Then, each composite rhizosphere soil sample was divided into three parts evenly, and a portion of which was air-dried for two weeks and stored at room temperature prior to nutrient analysis. Other two parts of these rhizosphere soil samples were immediately frozen in liquid nitrogen and then stored at 4°C and −80°C in a refrigerator for analyses of enzyme activity and microbe, respectively.

### Measurement of soil nutrients

The rhizosphere soil nutrients were measured as described by Yang et al. [32]. The organic matter was assayed using the potassium dichromate dilution thermal method. Total N was determined using the Kjeldahl distillation method. Total P was measured using the molybdenum-antimony-D-isoascorbic acid colorimetry after wet digestion with HClO_4_-H_2_SO_4_. Total K was determined using the flame photometry after molten with NaOH. Available N was assayed using the alkaline hydrolysis diffusion method. Available P was extracted with 0.5 mol L^−1^ NaHCO_3_ solution and then measured using the molybdenum-antimony-D-isoascorbic acid colorimetry. Available K was extracted with 1 mol L^−1^ NH_4_OAc neutral extraction and then determined by the flame photometry.

### Measurement of soil enzyme activities

The catalase and polyphenol oxidase activities were assayed according to the method of Zhou et al. [33]. The urease activity was assayed according to the method of Wang et al. [34]. Catalase activity was assayed using the potassium permanganate titration method and one unit of enzyme activity (U) was defined as a consumption of KMnO_4_ solution in 1 mL after 30 min. Polyphenol oxidase activity was measured using the pyrogallol colorimetry method and one unit of enzyme activity (U) was defined as a formation of purple pyrogallol in 1 mg after 2 h. Urease activity was determined using the phenol-sodium hypochlorite colorimetry method and one unit of enzyme activity (U) was defined as a formation of NH_4_-N in 1 mg after 24 h.

### Determination of soil microbes

Rhizosphere soil microbes were determined through the phospholipid fatty acids (PLFAs) analysis according to the modification method of Luo et al. [35]. Briefly, 4 g of rhizosphere soil sample was extracted with 15.2 mL of extracting solution, which contained 3.2 mL of citric acid buffer, 4 ml of chloroform and 8 mL of methanol. The extracts were centrifuged at 4000 r min^−1^ for 10 min and the supernatants were evaporated under N_2_. Phospholipids were separated on silicic acid columns by sequentially eluting with organic solvents of increasing polarity, and then saponified and methylated to form fatty-acid methyl esters (FAMEs). FAMEs were dissolved with hexyl hydride and then quantified with 19:0 methyl ester internal standard using a gas chromatograph. Identification of FAMEs was conducted using Sherlock Microbial Identification System 4.5. Individual PLFAs were used to indicate broad groups of the microbe community as follow: 12:0, 13:0, a13:0, i13:0, 14:0, a14:0, i14:0, a15:0, i15:0, i15:1G, 16:0, a16:0, i16:0, i16:1H, 16:02OH, 16:1ω5c, 16:1ω11c, 17:0, a17:0, i17:0, cy17:0, 17:1ω8c, 18:0, 11Me18:1ω7c, a19:0, i19:0, cy19:0ω8c, and 20:0 for bacteria [32, 36]; a16:0, 16:02OH, 16:1ω5c, 17:0, cy17:0, 17:1ω8c, 18:0, and cy19:0ω8c for gram-negative bacteria; 14:0, a14:0, i14:0, a13:0, a15:0, i15:0, i15:1G, i16:0, a17:0, and i17:0 for gram-positive bacteria; 18:1ω9c and 20:1ω9c for fungus [35, 37]; 10Me17:0 and 10Me18:0 for actinomycetes [38].

### Data analysis

One-way analysis of variance (ANOVA) was performed using DPS v7.05 software, and means were tested by least significant difference at the *P* < 0.05 level (LSD_0.05_). Figures were drawn by SigmaPlot 12.5 software. Pearson correlation coefficient and multiple stepwise regression equation were used to reflect the relationships between soil microbes and soil nutrients as well as soil enzyme activities.

## Results

### Rhizosphere soil nutrients of waxy sorghum

**Fig 1:**
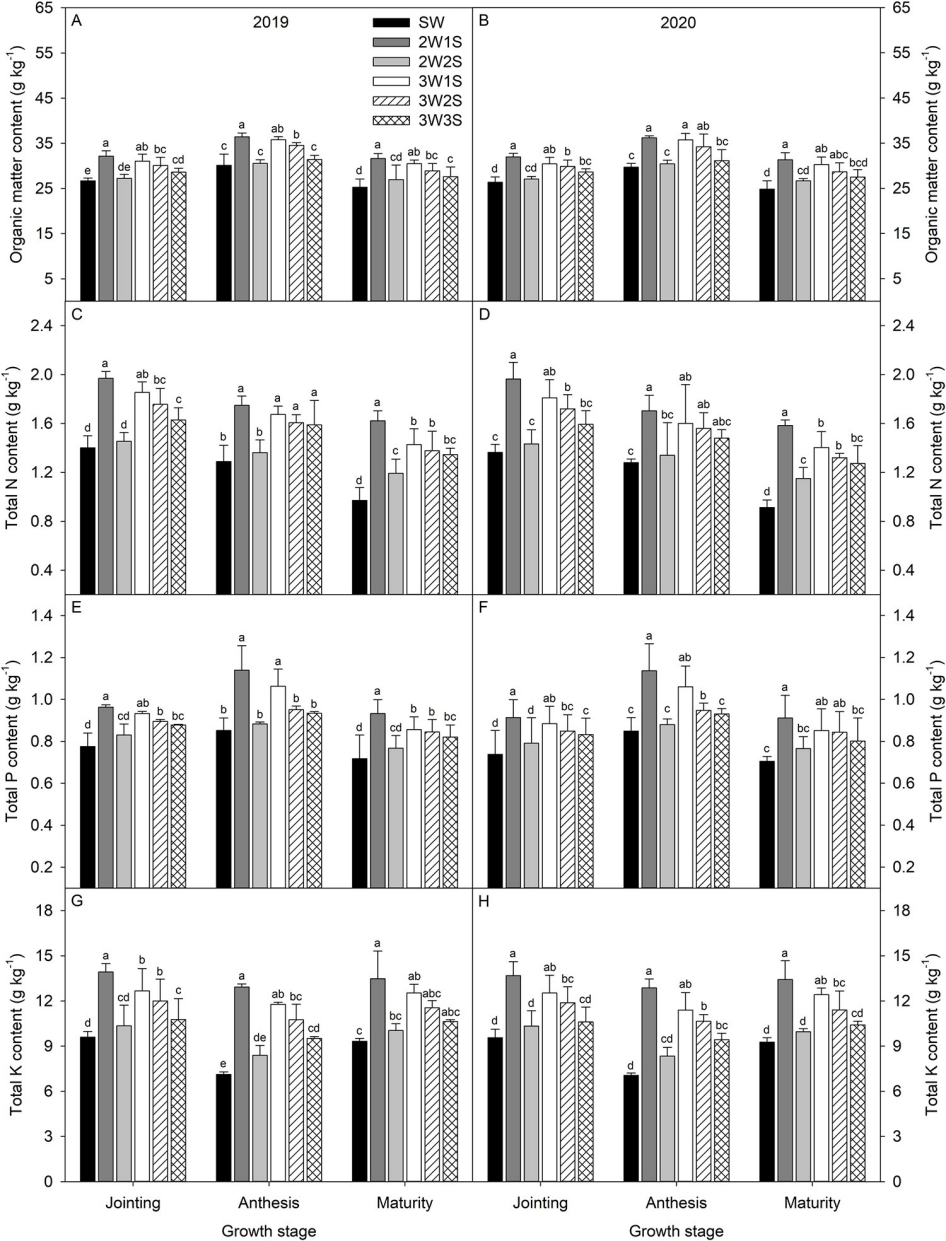
Rhizosphere soil organic matter (A, B), total N (C, D), total P (E, F), and total K (G, H) contents of waxy sorghum under different row ratio configurations in waxy sorghum-soybean intercropping systems. Data are expressed as mean o± *SE* (*n* = 3). Different letters indicate significant differences among treatments at the *P* < 0.05 level. SW represent sole cropping waxy sorghum. 2W1S, 2W2S, 3W1S, 3W2S, and 3W3S represent two rows of waxy sorghum intercropped with one row of soybean, two rows of waxy sorghum intercropped with two rows of soybean, three rows of waxy sorghum intercropped with one row of soybean, three rows of waxy sorghum intercropped with two rows of soybean, and three rows of waxy sorghum intercropped with three rows of soybean, respectively.

From jointing stage to maturity stage, the organic matter (Fig 1A and 1B) and total P contents (Fig 1E and 1F) increased and the decreased, total N content (Fig 1C and 1D) decreased gradually, while total K content (Fig 1G and 1H) decreased and then increased. Among all treatments, the performances of organic matter, total N, total P, and total K contents were 2W1S > 3W1S > 3W2S > 3W3S > 2W2S > SW in each growth stage. Compared to SW treatment, the 2W1S treatment increased the organic matter, total N, total P, and total K contents by 20.86%, 42.25%, 23.98%, and 44.12% at the jointing stage, 21.42%, 34.33%, 33.83%, and 81.86% at the anthesis stage, and 25.67%, 70.05%, 29.58%, and 44.68% at the maturity, respectively.

**Fig 2:**
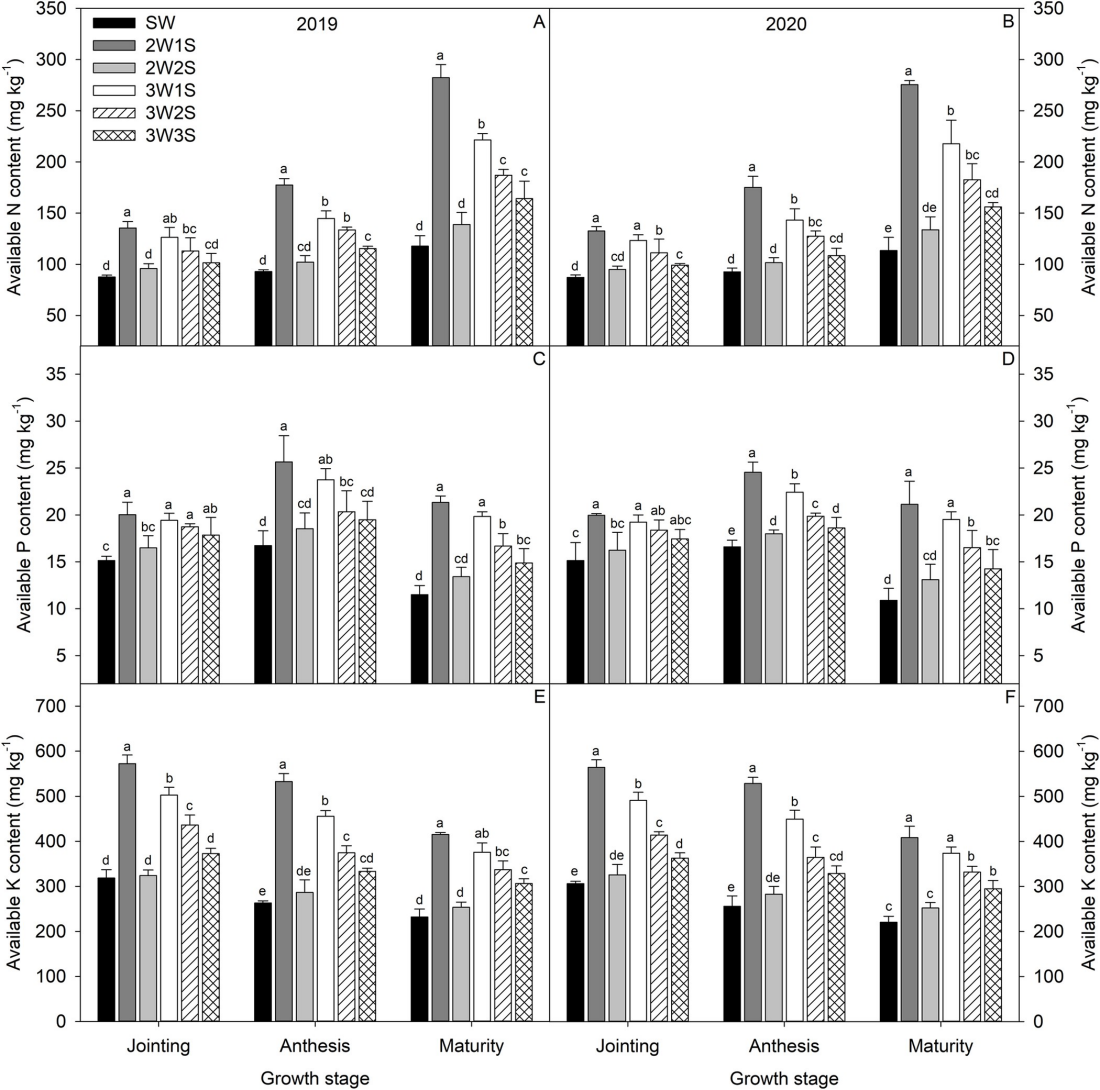
Rhizosphere soil available N (A, B), available P (C, D), and available K (E, F) contents of waxy sorghum under different row ratio configurations in waxy sorghum-soybean intercropping systems. Data are expressed as mean ± *SE* (*n* = 3). Different letters indicate significant differences among treatments at the *P* < 0.05 level. SW represent sole cropping waxy sorghum. 2W1S, 2W2S, 3W1S, 3W2S, and 3W3S represent two rows of waxy sorghum intercropped with one row of soybean, two rows of waxy sorghum intercropped with two rows of soybean, three rows of waxy sorghum intercropped with one row of soybean, three rows of waxy sorghum intercropped with two rows of soybean, and three rows of waxy sorghum intercropped with three rows of soybean, respectively.

The dynamic variation trend with growth stage for available N content (Fig 2A and 2B) was increased gradually, available P content (Fig 2C and 2D) was increased and the decreased, and available K content (Fig 2E and 2F) was decreased gradually from jointing stage to maturity stage. Similarly, the available N, available P, and available K contents under all row ratio configurations of waxy sorghum intercropped soybean were higher than those of sole cropping waxy sorghum, and the 2W1S treatment showed the best performance. The available N, available P, and available K contents under the 2W1S treatment were 1.53, 1.32, and 1.82 times at the jointing stage, 1.90, 1.51, and 2.05 times at the anthesis stage, and 2.41, 1.89, and 1.82 times at the maturity stage than those of SW treatment, respectively.

### Rhizosphere soil enzymes activities of waxy sorghum

**Fig 3:**
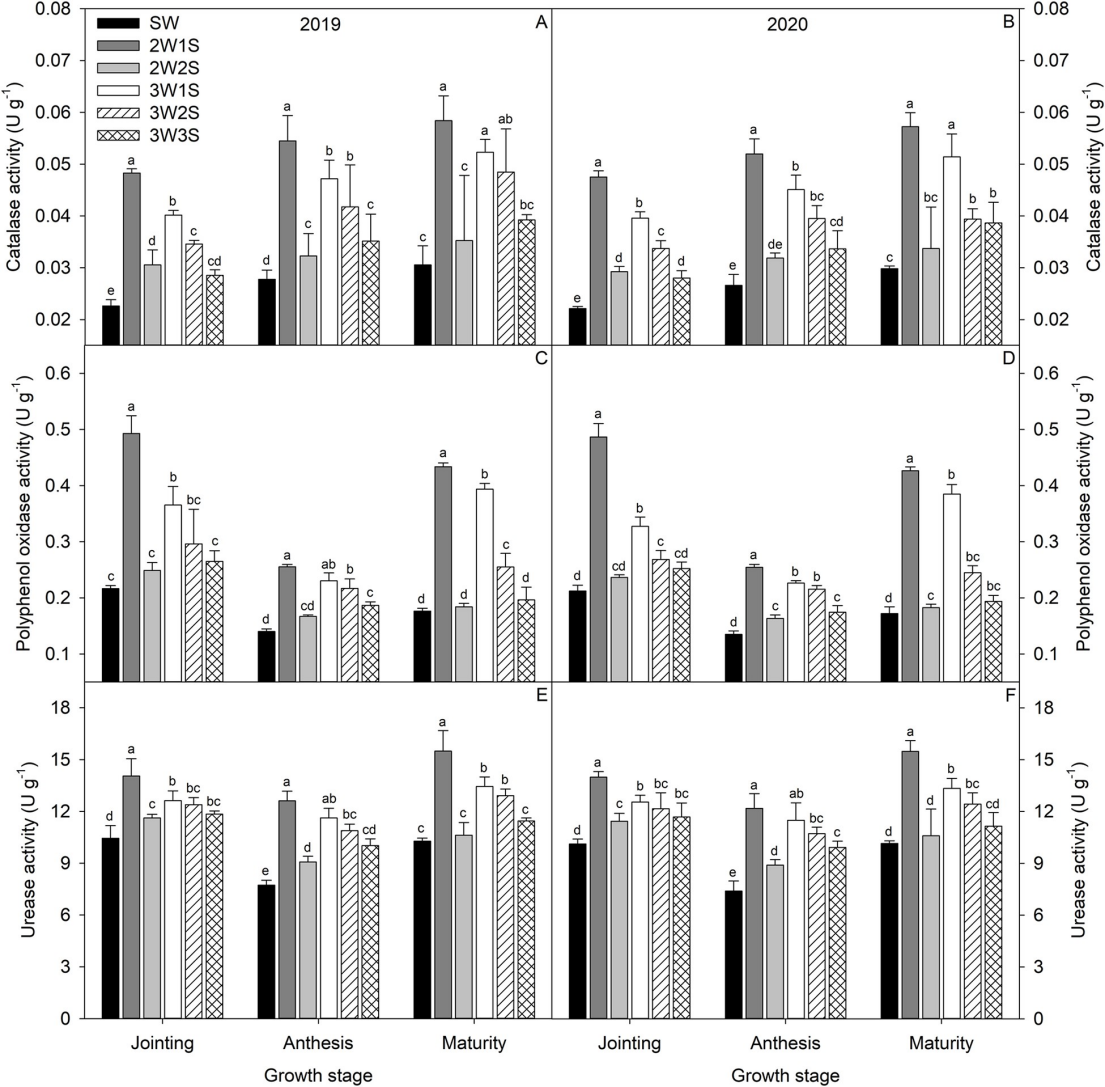
Rhizosphere soil catalase (A, B), polyphenol oxidase (C, D), and urease (E, F) activities of waxy sorghum under different row ratio configurations in waxy sorghum-soybean intercropping systems. Data are expressed as mean ± *SE* (*n* = 3). Different letters indicate significant differences among treatments at the *P* < 0.05 level. SW represent sole cropping waxy sorghum. 2W1S, 2W2S, 3W1S, 3W2S, and 3W3S represent two rows of waxy sorghum intercropped with one row of soybean, two rows of waxy sorghum intercropped with two rows of soybean, three rows of waxy sorghum intercropped with one row of soybean, three rows of waxy sorghum intercropped with two rows of soybean, and three rows of waxy sorghum intercropped with three rows of soybean, respectively.

From jointing stage to maturity stage, the dynamic variation trend for catalase activity (Fig 3A and 3B) was increased gradually, and polyphenol oxidase (Fig 3C and 3D) and urease activities (Fig 3E and 3F) were decreased and then increased. Among all treatments, the performances of catalase, polyphenol oxidase, and urease activities were 2W1S > 3W1S > 3W2S > 3W3S > 2W2S > SW in each growth stage. Compared to SW treatment, the 2W1S treatment increased the catalase, polyphenol oxidase, and urease activities by 114.07%, 128.65%, and 36.32% at the jointing stage, 95.77%, 85.35%, and 63.94% at the anthesis stage, and 91.44%, 146.91%, and 51.58% at the maturity, respectively.

### Rhizosphere soil microbes of waxy sorghum

**Fig 4:**
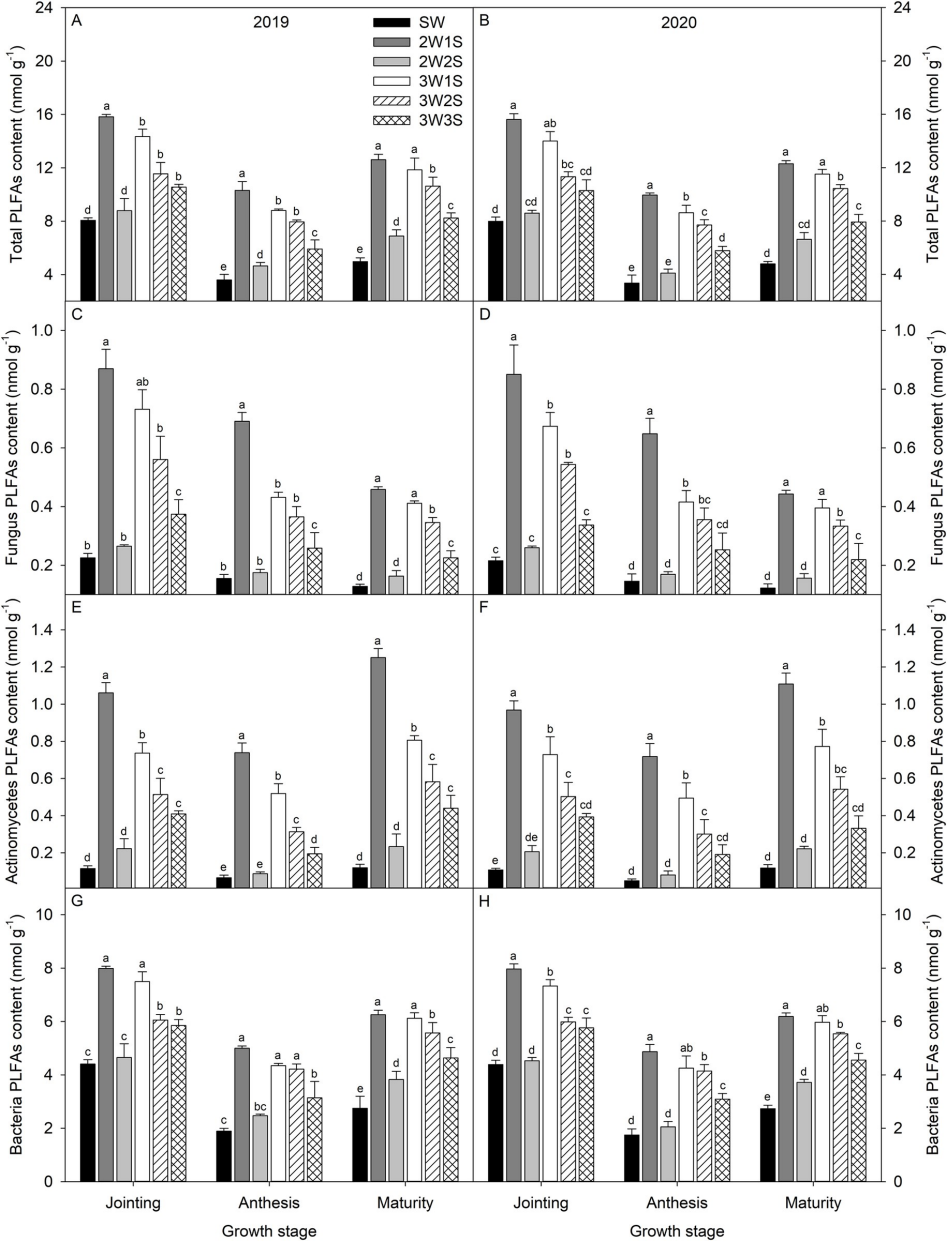
Rhizosphere soil total PLFAs (A, B), fungus PLFAs (C, D), actinomycetes PLFAs (E, F), and bacteria PLFAs (G, H) contents of waxy sorghum under different row ratio configurations in waxy sorghum-soybean intercropping systems. Data are expressed as mean ± *SE* (*n* = 3). Different letters indicate significant differences among treatments at the *P* < 0.05 level. SW represent sole cropping waxy sorghum. 2W1S, 2W2S, 3W1S, 3W2S, and 3W3S represent two rows of waxy sorghum intercropped with one row of soybean, two rows of waxy sorghum intercropped with two rows of soybean, three rows of waxy sorghum intercropped with one row of soybean, three rows of waxy sorghum intercropped with two rows of soybean, and three rows of waxy sorghum intercropped with three rows of soybean, respectively.

The dynamic variation trend with growth stage for total PLFAs (Fig 4A and 4B), actinomycetes PLFAs (Fig 4E and 4F), and bacteria PLFAs contents (Fig 4G and 4H) were decreased and then increased, while fungus PLFAs content (Fig 4C and 4D) was decreased gradually. The total PLFAs, fungus PLFAs, actinomycetes PLFAs, and bacteria PLFAs contents under all row ratio configurations of waxy sorghum intercropped soybean were higher than those of sole cropping waxy sorghum, and the 2W1S treatment showed the best performance. The total PLFAs, fungus PLFAs, actinomycetes PLFAs, and bacteria PLFAs contents under the 2W1S treatment were 1.96, 3.90, 9.11, and 1.81 times at the jointing stage, 2.91, 4.44, 12.56, and 2.71 times at the anthesis stage, and 2.55, 3.59, 9.91, and 2.27 times at the maturity stage than those of SW treatment, respectively.

Similarly, the gram-negative bacteria PLFAs and gram-positive bacteria PLFAs contents (Table 1) decreased and then increased with the growth process of waxy sorghum. The performances of gram-negative bacteria PLFAs and gram-positive bacteria PLFAs contents among all treatments were 2W1S > 3W1S > 3W2S > 3W3S > 2W2S > SW in each growth stage. Compared to SW treatment, the 2W1S treatment increased the gram-negative bacteria PLFAs and gram-positive bacteria PLFAs contents by 74.87% and 81.59% at the jointing stage, 194.32% and 136.59% at the anthesis stage, and 190.95% and 106.94% at the maturity stage, respectively.

**Table 1:** Rhizosphere soil gram-negative bacteria and gram-positive bacteria PLFAs contents of waxy sorghum under different row ratio configurations in waxy sorghum-soybean intercropping systems.

Year	Treatment	GNB PLFAs content (nmol g^−1^)			GPB PLFAs content (nmol g^−1^)		
		Jointing stage	Anthesis stage	Maturity stage	Jointing stage	Anthesis stage	Maturity stage
2019	SW	1.76 ± 0.19 d	0.66 ± 0.13 b	0.84 ± 0.24 c	1.56 ± 0.03 e	0.83 ± 0.05 d	1.14 ± 0.08 c
	2W1S	3.09 ± 0.11 a	1.94 ± 0.12 a	2.34 ± 0.21 a	2.82 ± 0.15 a	1.94 ± 0.11 a	2.31 ± 0.12 a
	2W2S	2.01 ± 0.09 cd	0.89 ± 0.16 b	1.14 ± 0.12 c	1.64 ± 0.07 de	1.02 ± 0.07 d	1.52 ± 0.18 bc
	3W1S	2.95 ± 0.12 ab	1.86 ± 0.23 a	2.29 ± 0.18 ab	2.43 ± 0.14 b	1.65 ± 0.12 b	2.22 ± 0.15 a
	3W2S	2.47 ± 0.22 bc	1.72 ± 0.12 a	2.02 ± 0.20 b	1.95 ± 0.07 c	1.34 ± 0.10 c	2.11 ± 0.13 a
	3W3S	2.13 ± 0.16 cd	1.06 ± 0.05 b	1.28 ± 0.31 c	1.79 ± 0.02 cd	1.27 ± 0.05 c	1.65 ± 0.16 b
2020	SW	1.75 ± 0.11 d	0.63 ± 0.17 c	0.76 ± 0.05 c	1.52 ± 0.13 c	0.78 ± 0.08 d	1.06 ± 0.10 c
	2W1S	3.05 ± 0.04 a	1.85 ± 0.19 a	2.31 ± 0.06 a	2.78 ± 0.08 a	1.86 ± 0.12 a	2.24 ± 0.04 a
	2W2S	1.97 ± 0.09 cd	0.84 ± 0.07 c	1.07 ± 0.11 bc	1.62 ± 0.13 c	0.96 ± 0.16 cd	1.46 ± 0.08 b
	3W1S	2.90 ± 0.15 a	1.83 ± 0.30 a	2.25 ± 0.24 a	2.36 ± 0.11 b	1.63 ± 0.17 a	2.13 ± 0.09 a
	3W2S	2.40 ± 0.10 b	1.62 ± 0.12 ab	1.95 ± 0.22 a	1.89 ± 0.16 c	1.29 ± 0.02 b	2.07 ± 0.13 a
	3W3S	2.06 ± 0.06 c	1.03 ± 0.29 bc	1.20 ± 0.16 b	1.73 ± 0.14 c	1.22 ± 0.14 bc	1.63 ± 0.14 b

Data are expressed as mean ± *SE* (*n* = 3). Different letters indicate significant difference among treatments at the *P* < 0.05 level. SW represent sole cropping waxy sorghum. 2W1S, 2W2S, 3W1S, 3W2S, and 3W3S represent two rows of waxy sorghum intercropped with one row of soybean, two rows of waxy sorghum intercropped with two rows of soybean, three rows of waxy sorghum intercropped with one row of soybean, three rows of waxy sorghum intercropped with two rows of soybean, and three rows of waxy sorghum intercropped with three rows of soybean, respectively.

### Relationships between soil microbes and soil nutrients as well as soil enzyme activities

As shown in correlation analysis (Table 2), soil microbes were significantly correlated with soil nutrients and soil enzyme activities except for organic matter and total P contents with total PLFAs, bacteria PLFAs, gram-negative bacteria PLFAs, and gram-positive bacteria PLFAs contents, and available N content with fungus PLFAs and gram-negative bacteria PLFAs contents. Further, multiple stepwise regression analysis (Table 3) showed that the determining factors of soil microbes were total K, catalase, and polyphenol oxidase for total microbes, bacteria, and gram-negative bacteria, total P and available K for fungus, available N, available K, and polyphenol oxidase for actinomycetes, and total K and polyphenol oxidase for gram-positive bacteria.

**Table 2:** Correlation coefficients between soil microbes and soil nutrients as well as soil enzyme activities.

Traits	Total PLFAs content	Fungus PLFAs content	Actinomycetes PLFAs content	Bacteria PLFAs content	Gram-negative bacteria PLFAs content	Gram-positive bacteria PLFAs content
Organic matter content	0.274	0.581 **	0.449 **	0.191	0.293	0.225
Total N content	0.756 **	0.894 **	0.647 **	0.719 **	0.798 **	0.676 **
Total P content	0.308	0.617 **	0.469 **	0.225	0.316	0.272
Total K content	0.926 **	0.853 **	0.931 **	0.896 **	0.873 **	0.928 **
Available N content	0.445 **	0.276	0.765 **	0.404 *	0.319	0.520 **
Available P content	0.449 **	0.673 **	0.592 **	0.367 *	0.478 **	0.401 *
Available K content	0.818 **	0.973 **	0.774 **	0.766 **	0.822 **	0.774 **
Catalase activity	0.594 **	0.609 **	0.864 **	0.527 **	0.493 **	0.643 **
Polyphenol oxidase activity	0.911 **	0.785 **	0.912 **	0.884 **	0.871 **	0.914 **
Urease activity	0.893 **	0.698 **	0.929 **	0.874 **	0.829 **	0.913 **

* and ** indicate significantly correlation at the *P* < 0.05 and *P* < 0.01 level, respectively.

**Table 3:** Multiple stepwise regression equations between soil microbes and soil nutrients as well as soil enzyme activities.

Multiple stepwise regression equation	Statistic	*R* ^2^	*F*	*P*
*y*_1_ = -7.41+1.64*x*_4_-129.03*x*_8_+13.75*x*_9_	1.55	0.978	232.92	< 0.001
*y*_2_ = -0.12–0.50*x*_3_+0.002*x*_7_	2.34	0.986	582.37	< 0.001
*y*_3_ = -0.75+0.003*x*_5_+0.001*x*_7_+1.41*x*_9_	1.85	0.989	477.94	< 0.001
*y*_4_ = -3.48+0.90*x*_4_-85.16*x*_8_+6.62*x*_9_	1.48	0.969	161.87	< 0.001
*y*_5_ = -1.59+0.38*x*_4_-38.89*x*_8_+2.97*x*_9_	1.42	0.958	120.28	< 0.001
*y*_6_ = -0.63+0.16*x*_4_+2.36*x*_9_	1.33	0.954	167.13	< 0.001

*y*_1_, total PLFAs content; *y*_2_, fungus PLFAs content; *y*_3_, actinomycetes PLFAs content; *y*_4_, bacteria PLFAs content; *y*_5_, gram-negative bacteria PLFAs content; *y*_6_, gram-positive bacteria PLFAs content; *x*_3_, total P content; *x*_4_, total K content; *x*_5_, available N content; *x*_7_, available K content; *x*_8_, catalase activity; *x*_9_, polyphenol oxidase activity.

## Discussion

Soil is a complex dynamic environment, which plays an important role in ecosystem function [33, 39]. Soil nutrients are important indicators to evaluate soil quality, which have been widely used to reflect the land productivity and crop productive potential [40, 41]. Intercropping is a sustainable planting pattern, which plays a crucial role in alleviating the land conflict between crops in Southwest China, especially in Guizhou province [30, 42]. A large number of studies have reported that intercropping improved soil nutrient content, biological activity and stability, increased soil fertilizer supply capacity, improved soil nutrient absorption environment, and ultimately promoted crop growth [15, 22, 27]. Gong et al. [43] reported that proso millet intercropped with mung bean obviously increased the total N, total P, total K, available P, and available K contents of proso millet rhizosphere soil. Similarly, in this study, waxy sorghum intercropped with soybean significantly affected the rhizosphere soil nutrients, and the total N, total P, total K, available N, available P, and available K contents of waxy sorghum rhizosphere soil under all row ratio configurations of waxy sorghum intercropped soybean were higher than those of sole cropping waxy sorghum. Soil organic matter is an important component of soil solid phase, which can improve soil physical properties, promote microbial activities and decomposition of nutrient elements in soil, and increase fertilizer preserving capability and buffer power of soil [32]. The present results showed that the performance of organic matter content among all treatments was 2W1S *>* 3W1S *>* 3W2S *>* 3W3S *>* 2W2S *>* SW, indicating that waxy sorghum intercropped with soybean can accelerate the accumulation of organic matter in waxy sorghum rhizosphere soil, which is similar to the previous study about green sword bean intercropped with waxy maize [44]. Thus, waxy sorghum intercropped with soybean can promote the accumulation of organic matter to improve nutritional status in waxy sorghum rhizosphere soil.

Soil enzymes are one of the components of soil, which participate in synthesis and decomposition of humus, hydrolysis and transformation of animals, plants, and microbes residues, and biochemical processes such as redox reactions of organic and inorganic compounds in soil [33, 45]. Soil catalase is a kind of oxidoreductase produced by plants or microbes, which plays an important role in the material circulation of soil ecosystem. It can catalyze the decomposition of hydrogen peroxide in soil into water and oxygen, so as to eliminate and reduce the harm of hydrogen peroxide [46]. Soil polyphenol oxidase is a compound enzyme that is decomposed and released by soil microbes, plant root exudates, and animal and plant residues, which can drive the decomposition and transformation of aromatic compounds in soil, eliminate the adverse effects of aromatic pollutants on soil quality, and thus complete soil environmental remediation [47]. Soil urease is an important part of soil ecosystem and plays an important role in soil material transformation and energy metabolism. As the driving force of soil metabolism, it can catalyze the hydrolysis of urea in soil to ammonia, and is an important index to evaluate the quality of ecological environment and soil fertility [48]. Gong et al. [43] demonstrated that proso millet intercropped with mung bean obviously increased the rhizosphere soil catalase and urease activities of proso millet. Zhou et al. [33] observed that the cucumber rhizosphere soil urease activity under cucumber-onion intercropping system and cucumber rhizosphere soil catalase activity under cucumber-garlic intercropping system were higher than that under sole cropping cucumber. Thus, it was not surprising to observe increases in catalase and urease activities of waxy sorghum rhizosphere soil were found under waxy sorghum-soybean intercropping system in the present study. However, Wu et al. [49] found that continuous sole cropping cucumber increased the polyphenol oxidase activity of cucumber rhizosphere soil, which does not in agreement with in the present study. This inconsistence might be due to the differences in soil ecosystems caused by crops themselves, as waxy sorghum belongs to the Gramineae family and cucumber belongs to the Cucurbitaceae family, which need to be further studied. These findings imply that waxy sorghum intercropped with soybean can increase enzymes activities and thus improve the quality of waxy sorghum rhizosphere soil.

Soil microbes are an important part of soil ecosystem, and their community composition and quantity changes reflect soil quality and health to a certain extent, and are also the key factors to overcome continuous cropping obstacles [50, 51]. Previous researches showed that rhizosphere soil microbes of crops were significantly affected by intercropping [33, 43, 52]. Therefore, a study on waxy sorghum rhizosphere soil microbes in different row ratio configurations of waxy sorghum intercropped with soybean is necessary to gain a high land use efficiency. PLFAs are present in the membranes of living cells but not in dead cells because they degrade rapidly during cell death, which can accurately estimate the microbial community structure in the rhizosphere soil [53]. In this study, waxy sorghum intercropped with soybean significantly affected the rhizosphere soil total PLFAs content of waxy sorghum and the total PLFAs content of waxy sorghum rhizosphere soil under all row ratio configurations was higher than that of sole cropping waxy sorghum. As far as we know, this is the first time to demonstrate the potential of wax sorghum-soybean intercropping system to regulate soil microbe populations in Guizhou Karst Mountains ecosystems. The present results showed that all row ratio configurations of waxy sorghum intercropped with soybean possessed higher fungus, actinomycetes, bacteria, gram-negative bacteria, and gram-positive bacteria PLFAs contents of waxy sorghum rhizosphere soil as compared to sole cropping waxy sorghum, which are consistent with previous findings on sugarcane-soybean [54], maize-peanut [55], and corn-soybean [56] intercropping systems. However, Zhou et al. [57] stated that Chinese milk vetch intercropped rape significantly reduced microbes contents in rape rhizosphere soil, including total PLFAs, bacteria, fungus, actinomycetes, gram-negative bacteria, and gram-positive bacteria. This difference might be due to the different accumulation of crop root exudates in the soil caused by the intercropping of crops at different family levels. In the present study, the bacteria PLFAs content under the same growth stage and treatment was the highest among the total PLFAs biomarkers in waxy sorghum rhizosphere soil. One possible reason for this phenomenon might be that the soil bacteria community was more sensitive to the effect of waxy sorghum intercropped with soybean [33]. Another explanation is that waxy sorghum intercropped with soybean promoted the waxy sorghum roots to produce and excrete more organic acids into rhizosphere soil [57]. The present study showed that the gram-negative bacteria and gram-positive bacteria PLFAs contents were roughly the same, suggesting that the nitrogen and carbon cycles of waxy sorghum rhizosphere soil reached an equilibrium state in waxy sorghum intercropped with soybean [56], which requires further studied. This finding demonstrates that waxy sorghum intercropped with soybean can promote the growth and diversity of waxy sorghum rhizosphere soil microbes, and thereby can enhance the soil health status in Guizhou Karst Mountains ecosystems.

## Conclusions

Rhizosphere soil properties of waxy sorghum were significantly affected by row ratio configurations of waxy sorghum intercropped soybean. In conclusion, 2W1S treatment was the optimal row ratio configuration of waxy sorghum intercropped with soybean, which can improve the rhizosphere soil quality and promote the sustainable production of waxy sorghum. Future studies will focus on soil microbial diversity based on high-throughput sequencing to better understand the changes in rhizosphere soil microenvironment of waxy sorghum.
